# Redox-Sensitive Fluorescent Nanoparticles for Biovisualization of Malignant Tumors

**DOI:** 10.17691/stm2025.17.1.05

**Published:** 2025-02-28

**Authors:** O. Peltek, E.A. Kopoleva, M.V. Zyuzin

**Affiliations:** Junior Researcher, Physics Department; ITMO University, 49, Bldg. A, Kronverksky Pr., Saint Petersburg, 197101, Russia; Engineer, Physics Department; ITMO University, 49, Bldg. A, Kronverksky Pr., Saint Petersburg, 197101, Russia; DSc, Leading Researcher; ITMO University, 49, Bldg. A, Kronverksky Pr., Saint Petersburg, 197101, Russia

**Keywords:** redox-sensitive nanoparticles, biovisualization, trithiocyanuric acid

## Abstract

Application of fluorescent redox-sensitive nanoparticles in current biomedicine ensures high sensitivity and accuracy of biovisualization. Nanoparticles are potent as they can long circulate in the blood, where the level of glutathione is relatively low, and are destroyed in tumor cells, releasing loaded dyes or drugs.

**The aim of the study** was to develop new nanoparticles based on trithiocyanuric acid for biovisualization of malignant tumors and study capabilities of the developed nanoparticles.

**Materials and Methods:**

Nanoparticles were obtained by polycondensation of trithiocyanuric acid using iodine. Scanning and transmission electron microscopy was used for their characterization, the loading of fluorescent dyes was assessed by means of spectrophotometry. Confocal laser scanning microscopy was applied to study the impact of nanoparticles on the viability of the 4T1 and A549 cell lines as well as their interaction with cells. The distribution of nanoparticles in tissues and organs of BALB/c model mice with grafted tumors was performed using fluorescence visualization.

**Results:**

According to scanning microscopy, the size of the synthesized particles reached 100±20 nm. The adsorption isotherm demonstrated that adsorption of 0.27 mg of the RhB fluorescent dye per 1 mg of nanoparticles could be achieved. Enhanced release of the packed fluorescent dye was seen in the presence of glutathione and acetylcysteine. The particles did not significantly affect the viability of 4T1 and A549 cells. After intratumoral administration, they ensured a more intense fluorescent signal in the tumor area compared to a regular fluorescent dye solution.

**Conclusion:**

The developed system of trithiocyanuric-acid-based nanoparticles demonstrated high efficiency in biovisualization of malignant tumors and has a potential for targeted delivery of treatment agents.

## Introduction

Fluorescent nanoparticles have an important position in present-day biomedicine as they can provide high sensitivity and accuracy of biovizualization [1]. They are actively used to study cellular structures [2], monitor biomolecules [3], deliver drugs [4, 5], and diagnose tumors [6]. The use of nanoparticles is of particular importance in biovizualization of tumor tissues. They can specify the tumor boundaries during surgical interventions, which allows the surgeon to more accurately remove tumor tissue thus decreasing damage to healthy tissues to the minimum [7–9]. This task can be implemented more effectively subject to nanoparticles having selectivity for tumor tissues and being able to stay in such tissues for a long time [10].

At present, biovizualization employs various types of nanoparticles including quantum dots [11], gold nanoparticles [12], magnetic iron oxide nanoparticles [13], carbon dots [14], and lanthanide nanoparticles [15]. Organic nanoparticles that can be functionally loaded with near-infrared fluorescent dyes are also of particular interest [16]. Redox-sensitive systems having unique properties are especially understudied compared to other organic nanoparticles.

Redox-sensitive nanoparticles are potent as they can long circulate in the blood, where the level of glutathione is relatively low, and are destroyed in tumor cells, releasing loaded dyes or drugs [17]. These properties allow the particles to be used both for targeted delivery of treatment agents and for improved visualization of tumor cells. However, despite the promising future outlook, the studies of redox-sensitive nanoparticles for biovisualization remain limited.

Development of such nanoparticles with improved properties can greatly benefit from studying of new materials with unique chemical structures and features. In the present research, trithiocyanuric acid (TTCA) was chosen as the basis for nanoparticles. Its structure includes disulfide groups thus ensuring redox sensitivity and allowing stable organic nanoparticles. Currently, TTCA has not yet been used to build nanoparticles for biovizualization.

**The aim of the study** was to synthesize, characterize, and study TTCA-based nanoparticles.

The capacity of nanoparticles to retain and release fluorescent agents, as well be accumulated in tumor tissues was assessed.

This study is intended to facilitate improvement of the knowledge base about the use of new materials in biomedicine, which allows developing more effective systems for tumor diagnosis and treatment.

## Materials and Methods

### Synthesis of nanoparticles

Nanoparticles were synthesized using the following reagents by Sigma- Aldrich, USA, which were used without additional purification: TTCA hydrate of sodium salt (98%), polyethyleneglycol diglycidyl ether (PEGDE, %), iodine (I_2_, 99%), and sodium iodide (NaI).

First, a 0.155 M solution of I_2_ in a 0.3875 M aqueous solution of NaI was prepared with a molar ratio of I2:NaI equal to 1:2.5. Then 345 μl of 0.155 M PEGDE solution were mixed with 60 μl of freshly prepared 2 M TTCA solution and 2107 μl of deionized water were added to the resulting solution. The solution was stirred for 10 min. The earlier prepared 0.155 M I_2_ solution in NaI solution was added to the TTCA solution in portions (5 portions in total) with 10 s intervals subject to intensive stirring (700 rpm). After additional stirring for 10 min, the new mixture turned pale white. Here, the synthesis process was completed, and the resulting nanoparticles were centrifuged at 14,000 rpm for 5 min and washed twice with water.

### Characterization of nanoparticles

The hydrodynamic radius of the synthesized nanoparticles was determined using a Zetasizer Nano ZS90 analyzer (Malvern, USA) equipped with a He-Ne laser with a power of 4.0 mW and a wavelength of 633 nm. For measurements, 100 μl of the synthesized nanoparticle solution were diluted in 1 ml of deionized water, and measurements were conducted in 40 μl cuvettes.

The synthesized TTCA-based nanoparticles were studied by scanning electron microscopy (SEM) using a TESCAN MIRA 3 scanning electron microscope (Tescan, the Czech Republic) with an accelerating voltage of 0.5– 30.0 keV. One day before the measurements, 2 μl of the TTCA-based nanoparticle suspension in water were applied to a silicon substrate and left until dry.

Transmission electron microscopy (TEM) images of nanoparticles were obtained using a Tecnai G2 F20 X-TWIN microscope (FEI, the Netherlands) equipped with a Gatan Orius CCD camera (Gatan Inc., USA). Measurements were performed in bright field mode at an accelerating voltage of 200 kV.

### Loading of fluorescent dye

Rhodamine B (RhB) was used as the model fluorescent dye. For dye adsorption, 0.75 mg of TTCA-based nanoparticles were mixed with 100 μl of the dye aqueous solution of different concentrations (0.25, 0.50, 0.75, 1.0) and diluted with water to a volume of 1 ml. Then the resulting solutions were stirred for 30 min and centrifuged for 5 min at 14,000 rpm. The residual supernatant containing unabsorbed RhB dye was measured spectrophotometrically at a wavelength of 552 nm. To calculate the amount of adsorbed RhB dye (in percent), the absorbance of pure TTCA-based nanoparticles supernatant was used as the background.

To assess the desorption and release of the loaded fluorescent dye in various solutions, 0.5 mg of TTCAbased nanoparticles with adsorbed RhB were diluted separately in 1 ml of water, dimethyl sulfoxide (DMSO), 95% ethyl alcohol, 250 mM acetylcysteine or 250 mM glutathione and stirred for 60 min. Then, nanoparticles were centrifuged for 5 min at 14,000 rpm. The amount of RhB dye released from the nanoparticles into the supernatant was determined spectrophotometrically by its absorbance at the wavelength of 552 nm. The amount of released RhB (in percent) was calculated by using the absorbance of TTCA-based nanoparticles supernatant incubated in the corresponding solvent as the background.

### Assessment of nanoparticles impact on cell viability

To assess the impact of TTCA-based nanoparticles on the viability of the 4T1 and A549 cell cultures, the cells were seeded in 96-well plates at 5.0×10^5^ cells per well. In two days, TTCAbased nanoparticles were added to the cells at final concentrations of 0.25, 0.5, 1, 2, and 4 mg/ml (in 200 μl of the medium). Then, the cells were incubated for 48 h. Further, living cells were stained with the Calcein-AM vital dye. The samples were washed twice with PBS buffer solution, and then 10 mM Calcein-AM solution in 200 μl of PBS was added. After 15 min, the stained cells were visualized using a Zeiss LSM 710 confocal laser scanning microscope (Carl Zeiss, Germany). Images were obtained using a 10× lens.

### Assessment of nanoparticle association with cells

To visualize the interaction of TTCA-based nanoparticles with 4T1 and A549 cells, the cells were seeded on Petri dishes for confocal microscopy (d=35 mm; Eppendorf, Germany) at a concentration of 5.0×10^5^ cells per dish. On the next day, TTCA-based nanoparticles loaded with the fluorescent dye RhB were added to the cells at final concentrations of 0.25, 0.5, 1, 2, and 4 mg/ml in 2 ml of the cell medium, and the cells were left overnight. On the next day, the cells were washed twice with PBS, then 2 ml of formalin solution were added and placed in the refrigerator for 30 min. Further, the samples were again washed with PBS and incubated for 15 min in a solution of 0.1% Triton X-100 in PBS. The cells were stained with 10 mM fluorescein solution, which was added to the samples together with 1.5 ml PBS. After 15 min, the cells were visualized using a Zeiss LSM 710 confocal laser scanning microscope (Carl Zeiss, Germany). Images were obtained using 10× and 40× lenses.

### Assessment of malignant tumors visualization using nanoparticles

To create a model pathology, 4T1 breast carcinoma cells (CRL-2539) were used. The cells were trypsinized, then washed twice with cold PBS and resuspended in PBS to a concentration of 1×10^6^ cells/ml. Further, the cells were injected subcutaneously (50 μl with a concentration of 1×10^6^ cells/ml) into legs of BALB/c mice. Seven days after tumor formation, animals with a tumor of a sufficient size (approximately 0.05±0.01 cm^3^) were used for further experiments.

Biodistribution of TTCA-based nanoparticles was assessed by fluorescence imaging using the IVIS Lumina II system (PerkinElmer Inc., USA). The system was operated in epifluorescence mode with excitation and emission wavelengths of 640 and 690 nm, respectively. Then, the model animals were injected either free Cy5.5 dye or TTCA-based nanoparticles loaded with Cy5.5 dye (50 μl) directly into the tumor. Both injections contained the same concentration of Cy5.5 (50 μg/ml or 2.5 μg per injection). Mice were euthanized 6 h after the injection, which allowed detection and registration of fluorescent signals from the major body organs (heart, liver, spleen, lungs, kidneys) and the tumor.

## Results

### Assessment of the size and morphology of synthesized nanoparticles

**Figure 1.**
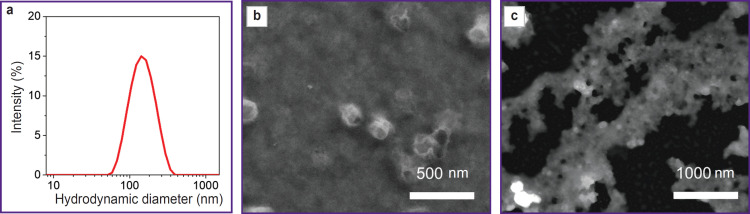
Properties of the obtained TTCA: (a) hydrodynamic diameter; (b) scanning electron microscopy; (c) transmission electron microscopy

The obtained TTCA-based nanoparticles were characterized using the dynamic light scattering method, SEM, and TEM (Figure 1).

The hydrodynamic diameter of the synthesized nanoparticles was 120±20 nm. According to SEM, their size was 100±20 nm.

### Assessment of the fluorescent dye loading

**Figure 2.**
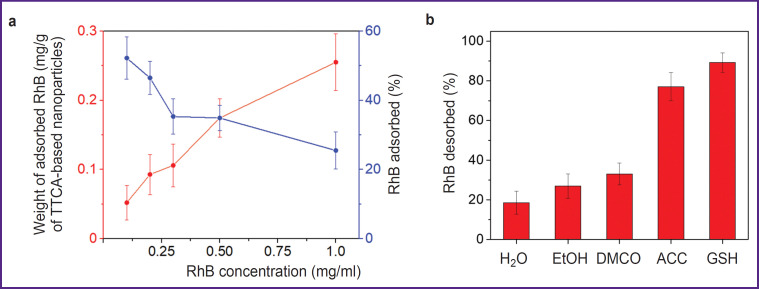
Loading and release of fluorescent dye: (a) weight and percentage of the adsorbed RhB dye depending on the initial concentration in the solution; (b) release of RhB dye from TTCA-based nanoparticles in various solvents. EtOH — ethyl alcohol, DMSO — dimethyl sulfoxide, ACC — acetylcysteine, GSH — glutathione

The adsorption isotherm showed that it was possible to get the adsorption of 0.27 mg of the fluorescent dye RhB per 1 mg of nanoparticles. At the RhB concentration of 1 mg/ml, approximately 25% of RhB from the solution was adsorbed. However, when this concentration was exceeded, the particles began to aggregate, thus, the adsorption was not assessed with higher values (Figure 2 (a)). In all subsequent experiments, nanoparticles were used with 0.2 mg of the RhB dye adsorbed per 1 mg of TTCA-based nanoparticles.

The assessment of the RhB dye desorption from TTCA-based nanoparticles incubated in various solvents (including acetylcysteine and glutathione solutions — 77.1 and 89.2%, respectively) was also conducted (Figure 2 (b)). The obtained values were due to the nanoparticles degradation as a result of the disulfide bonds cleavage in presence of acetylcysteine and glutathione. One should mention that the presence of glutathione or acetylcysteine caused accelerated release of the dye, unlike water, alcohol or DMSO, in which desorption occurred gradually over 60 min.

### The impact of nanoparticles on tumor cell viability

**Figure 3.**
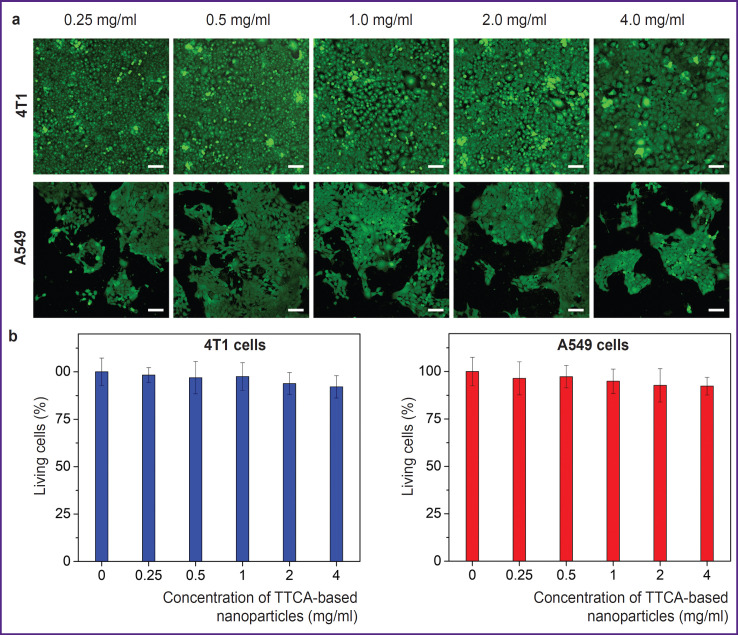
The impact of TTCA-based nanoparticles on the viability of 4T1 and A549 cells: (a) images of cells stained with the Calcein-AM vital dye, which were obtained using confocal laser scanning microscopy; bar is equal to 50 μm; (b) quantitative assessment of nanoparticle impact on cell viability

The assessment of the nanoparticles impact on tumor cell viability was conducted using two cell lines: 4T1 mouse breast cancer cells and A549 human lung carcinoma cells. These tumor cell lines were chosen due to their different intracellular glutathione content: A549 cells contain more glutathione than 4T1 cells [18, 19]. According to the results, TTCA-based nanoparticles at a concentration of maximum 4 mg/ml did not significantly impact the viability of either cell line. For example, the cell viability of A549 cells, at the highest concentration of TTCA-based nanoparticles (4 mg/ml) decreased only to 90% compared to the control (Figure 3).

### Assessment of nanoparticle association with tumor cells

**Figure 4.**
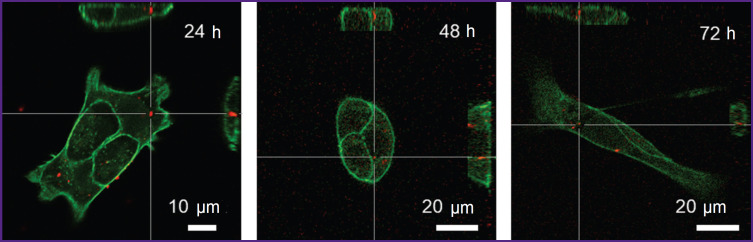
Images of 4T1 cells stained with fluorescein-labeled phalloidin Red fluorescence is seen from RhB-labeled TTCA-based nanoparticles. Images were obtained by means of confocal laser scanning microscopy at different incubation intervals

The nanoparticles association with tumor cells and their subsequent internalization were studied using the 4T1 cell line. To study this process in dynamics, the cells were visualized at different time intervals (24, 48, and 72 h). It was established that during this time, the RhB signal partially weakens, but specific particles were still distinguishable (Figure 4).

### Assessment of the TTCA-based nanoparticles biodistribution

**Figure 5.**
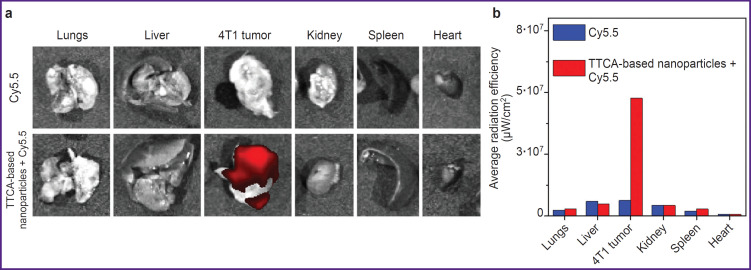
Assessment of the TTCA-based nanoparticles distribution in body organs of model animals after intratumoral administration: (a) photos of the major organs of the mice that were injected with either TTCA-based nanoparticles with Cy5.5 or Cy5.5 solution, 6 h after administration; (b) quantitative assessment of the Cy5.5 signal intensity from various organs

To study the nanoparticles biodistribution, the nanoparticles were loaded with Cy5.5 dye, which was chosen as a far-red fluorescent dye to visualize nanoparticles in tissues. The experiment was conducted on a BALB/c mouse model with 4T1 tumor. The tumor developed after subcutaneous administration of 4T1 cells. The distribution of TTCA-based nanoparticles loaded with Cy5.5 was assessed using fluorescence visualization. Comparison of the the results of the experiment with the dye loaded into TTCA and unloaded Cy5.5 demonstrated that 6 h after intratumoral administration the major part of nanoparticles remained in tumor tissues (Figure 5). Moreover, after 6 h, the nanoparticles demonstrated a significantly higher degree of Cy5.5 retention in the tumor tissues compared to the unloaded dye.

## Discussion

This study demonstrates a new system of redoxsensitive TTCA-based nanoparticles and looks into such nanoparticles potential for biovizualization. The study results prove that these nanoparticles have unique properties, which may be useful for biomedical applications. The synthesis of TTCA-based nanoparticles is reproducible and not complicated, which is an advantage for further research scaling up. The nanoparticles reached 120±20 nm, being an optimal range to benefit from the effect of increased permeability and retention in tumor tissues. The morphology of the particles, confirmed by means of SEM and TEM, indicates their stability, which is of significance for further biological experiments. It is worth mentioning that the disulfide groups within TTCA ensure redox sensitivity, but do not cause significant aggregation of particles, which is often a problematic issue of such systems.

Experiments demonstrated that TTCA-based nanoparticles can efficiently retain rhodamine B, with adsorption being optimal at the dye concentration of 1 mg/ml. In redox-active environments (such as glutathione or acetylcysteine), rhodamine B is significantly greater released than in neutral solvents. This confirms the selectivity of nanoparticle degradation in tumor tissues having increased glutathione levels. Such data highlight the potential of redox-sensitive systems for targeted delivery. Cytotoxicity of TTCAbased nanoparticles on 4T1 and A549 cells is insignificant even at the concentration of 4 mg/ml, thus showing high biocompatibility of the system. This is especially important for potential application in clinical practice, where minimized toxicity is a major requirement.

Confocal visualization showed that TTCA-based nanoparticles efficiently associate with cells and remain stable for 72 h, although the dye signal intensity decreases over time. This may be due to gradual metabolism of nanoparticles inside the cells and release of the dye.

Biodistribution in the 4T1 tumor model demonstrated that nanoparticles remained in tumor tissues 6 h after intratumoral administration, whereas free dye was rapidly cleared. This is a major result, confirming that TTCA-based nanoparticles provide stable accumulation in the tumor, which may be useful for diagnostics and treatment. Compared with other redox-sensitive nanoparticles, such as polysulfide polymers, the TTCAbased system is less toxic, whereas its synthesis is easier and more scalable. Finally, the examined nanoparticles have a high loading capacity.

One should note that regardless of the promising results, the study has some limitations. For example, biodistribution was studied only after intratumoral administration. Further, it is planned to study the pharmacokinetics after intravenous administration to assess the potential of using TTCA-based nanoparticles for systemic delivery. Another research line relates to modification of the nanoparticle surface to improve selectivity to tumor tissues.

## Conclusion

The new developed and characterized trithiocyanuric acid-based nanoparticle system demonstrated high efficiency in biovizualization and potentially in targeted delivery of treatment agents. The unique structure of TTCA, including disulfide groups, ensures redox sensitivity, thus making such nanoparticles particularly promising for selective destruction in tumor tissues. The obtained nanoparticles have an optimal size (120±20 nm) for accumulation in tumors due to increased permeability and retention. They demonstrated high stability, ability to retain and release fluorescent molecules over glutathione, as well as minimal toxicity to cells. Biodistribution showed that nanoparticles remained in the tumor tissue after intratumoral administration, whereas free dye was rapidly cleared.

The study demonstrates the potential of using such materials as TTCA to make biocompatible and functional nanoparticles. This empowers development of systems that can be used for both tumor diagnostics and treatment.
